# Editorial: Plant natural products: biosynthesis, regulation, and function

**DOI:** 10.3389/fpls.2025.1661170

**Published:** 2025-07-31

**Authors:** Zongxia Yu, Yongjun Wei, Boyang Ji, Xin Fang

**Affiliations:** ^1^ Jiangxi Key Laboratory for Sustainable Utilization of Chinese Materia Medica Resources, Lushan Botanical Garden, Jiangxi Province and Chinese Academy of Sciences, Jiujiang, China; ^2^ School of Pharmaceutical Sciences, State Key Laboratory of Antiviral Drugs, Pingyuan Laboratory, Zhengzhou University, Zhengzhou, China; ^3^ BioInnovation Institute, Copenhagen, Denmark; ^4^ State Key Laboratory of Phytochemistry and Natural Medicines in West China, Kunming Institute of Botany, Chinese Academy of Sciences, Kunming, China

**Keywords:** plant specialized metabolites, biosynthesis, metabolomics, medicinal plants, phytochemistry

Plant natural products, also referred to as plant specialized metabolites, are small molecules synthesized by plants. Throughout history, human have harnessed these compounds for diverse applications, including development of medicines, food supplements, and dyes. In their host organisms, these molecules perform diverse functions, such as mediating pollinators and mycorrhizal fungi interactions, defensing against biotic stresses like herbivores and pathogens, and protecting against abiotic stresses like UV-B radiation, frost, and drought (Dixon and Paiva, 1995).

Traditional phytochemical approaches, which typically involve isolating individual metabolites followed by targeted bioassays, have long provided foundational insights into the functions of these compounds. However, given the immense chemical diversity of plant natural products, estimated to exceed one million distinct structures (Afendi et al., 2012), these conventional methods are ill-suited for exploring the full metabolic landscape. The emergence of metabolomics has transformed this paradigm by enabling comprehensive, high-throughput analysis of hundreds to thousands of metabolites simultaneously, thereby offering unprecedented capacity to decipher metabolic functions. Moreover, these compounds are often minute quantities in planta, elucidating the biosynthetic pathways and regulatory networks that govern specialized metabolites is critical. Such understanding facilitates the development of synthetic biology strategies for heterologous production of these compounds in engineered organisms.

This Research Topic, entitled *“Plant Natural Products: Biosynthesis, Regulation, and Function,”* features 14 original research articles, one review, and one opinion piece from authors worldwide. Collectively, these contributions highlight cutting-edge discoveries across all aspects of plant specialized metabolism, from molecular mechanisms to ecological significance.

Metabolomics has emerged as a powerful tool for deciphering the dynamic chemical diversity of plants, offering insights into species-specific metabolites, developmental stage variations, and environmental responses. This approach is crucial due to the high specificity and contextual variability of plant natural products, necessitating comprehensive analyses across diverse species, ontogenetic stages, and environmental conditions. Pannequin et al. performed a comparative metabolome study of a large collection of bryophyte plants including 60 species, 15 orders, and 41 families, which provides a comprehensive overview of bryophyte chemodiversity and in-depth chemical feature of certain species. Liu et al. combined widely targeted metabolomics with biological activities assay, and sensory flavor analyses, revealing that the buds of *Lonicera japonica* Thunb. var. *chinensis* had great exploitation potentials in pharmaceuticals, beverages, and nutraceuticals. Yang et al. identified 154 common differential metabolites across diverse ripening stages of *Baccaurea ramiflora* Lour. fruit based on non-targeted metabolomics analysis, with L-sorbose and 5-hydroxyindole-3-acetic acid as taste biomarkers. Environmental stimuli frequently trigger plant natural products biosynthesis. Wang et al. investigated the effects of geography, soil and climatic factors on the two main secondary metabolites contents in the roots of *Rubia cordifolia* L. Their findings revealed that annual precipitation negatively correlated with the contents of purpurin and mollugin.

Notably, metabolomics combined with other omics approaches can provide an unbiased view of biological processes, thus facilitating the elucidation of underlying molecular mechanism. By integrated analyses of transcriptomic and metabolomic data from plant samples across various developmental stages and distinct locations, Chen et al. and Tian et al. uncovered the seasonal and spatial dynamics, as well as molecular regulation of flavonoid biosynthesis in *Cyclocarya paliurus* and *Epimedium sagittatum*, respectively. In another study, Yan et al., employed proteomic and metabolome analyses to reveal the reconfiguration of energy metabolism and terpenoid biosynthesis in cigar tobacco under low-light conditions, identifying 254 significantly differentially expressed metabolites and 780 significantly differentially expressed proteins.

Despite the rapid development of metabolomic technology, traditional phytochemical method remains playing a critical role in elucidating the functions of novel natural products. Liu et al. isolated six eudesmane-type sesquiterpenoids from *Laggera pterodonta*, and these compounds exhibited varying degrees of inhibitory effects against six plant pathogenic fungi, including *Phytophthora nicotianae*, *Fusarium oxysporum*, *Alternaria alternata*, *Gloeosporium frucrigenum* Berk, *Colletotrichum fructicola*, and *Botrytis cinerea*. Ruan et al. characterized nine new ent-atisane-type diterpenoids from *Euphorbia fischeriana* Steud, one of which exhibited significant antiviral activity against COVID-19 by directly binding to the virus’s RNA-dependent RNA polymerase. Additionally, Neel et al. reported that two major metabolites, gymnemic acid IV and gymnestrogenin from the leaves of *Gymnema sylvestre* were effective against *Penicillium digitatum* 6952, *Penicillium expansum* 2995, and *Aspergillus flavus* 6678.

In recent years, with the maturity of high-throughput sequencing technology and advancements in bioinformatics analysis methods, researches on the biosynthesis and regulation of natural products have expanded from model plants to medicinal plants. Wu et al. compared the function and sequences of key oil biosynthetic genes across different cultivars of *Perilla frutescens*, discovering crucial amino acid residues responsible for the catalytic activity of Δ12 fatty acid desaturases. Zeng et al. unveiled that the influence of sugar metabolism on oil synthesis varies throughout distinct fruit development stages in oil plant *Symplocos paniculata*. Senevirathne et al. summarized the mechanism of oxylipin signaling molecules to regulate phytocannabinoids production in *Cannabis sativa*. MYB transcription factors (TFs) are a class of important TFs that regulate the synthesis of various plant secondary metabolites. Wang et al. conducted a genome-wide analysis of MYB TFs in four *Rheum* L. plants, identifying 1054 MYB genes, with 12 characterizing a key role in anthraquinones biosynthesis. Virus-induced gene silencing (VIGS) is a powerful tool to detect gene functions *in vivo*, particularly in non-model plants. Yu et al. developed an easy and effective VIGS approach, which could facilitate endogenous gene studies in two *Nepeta* species. Finally, considering the complexity of plant secondary metabolism biosynthesis, Yin and Yang discussed an alternative chemoproteomic approach that uses affinity probes to identify active enzymes to elucidate biosynthetic pathway.

To date, our knowledge of the biosynthesis and regulation of specialized metabolites across various plant species remains limited. This Research Topic significantly advances the knowledge framework for integrated approach to understand the production and functions of plant natural products. We hope this Research Topic can serve as a landmark reference for future research in the field.
